# Nidogen-1, a Player in KMT2A-Rearranged Pediatric Acute Myeloid Leukemia

**DOI:** 10.3390/ijms26073011

**Published:** 2025-03-26

**Authors:** Jolien Vanhooren, Larissa Deneweth, Luca Pagliaro, Zhiyao Ren, Mariateresa Giaimo, Rafaella Zamponi, Giovanni Roti, Barbara Depreter, Mattias Hofmans, Barbara De Moerloose, Tim Lammens

**Affiliations:** 1 Department of Internal Medicine and Pediatrics, Ghent University, 9000 Ghent, Belgium; jolien.vanhooren@ugent.be (J.V.);; 2 Department of Pediatric Hematology-Oncology and Stem Cell Transplantation, Ghent University Hospital, 9000 Ghent, Belgium; 3 Cancer Research Institute Ghent (CRIG), 9000 Ghent, Belgium; 4 Translational Hematology and Chemogenomics (THEC), University of Parma, 43121 Parma, Italy; 5 Department of Medicine and Surgery, University of Parma, 43121 Parma, Italy; 6 Hematology and BMT Unit, Azienda Ospedaliero-Universitaria di Parma, 43121 Parma, Italy; 7 Department of Laboratory Medicine, AZ Delta General Hospital, 8800 Roeselare, Belgium; 8 Department of Haematology, Vrije Universiteit Brussel (VUB), 1000 Brussels, Belgium; 9 Department of Diagnostic Sciences, Ghent University, 9000 Ghent, Belgium; 10 Department of Laboratory Medicine, Ghent University Hospital, 9000 Ghent, Belgium

**Keywords:** AML, leukemic stem cell, nidogen-1

## Abstract

Despite advances in outcome, one third of children with acute myeloid leukemia (AML) relapse, and less than half will achieve long-term survival. Relapse in AML has been shown to be driven in part by leukemic stem cells (LSCs), highlighting the unmet medical need to better characterize and target this therapy-resistant cell population. Micro-array profiling of pediatric AML subpopulations (LSCs and leukemic myeloblasts) and their healthy counterparts revealed *nidogen-1* (NID1) as expressed in both leukemic subpopulations while absent in the hematopoietic stem cell. Using the TARGET dataset including pediatric AML patients (n = 1025), NID1 expression showed a correlation with worse event-free survival and KMT2A rearrangements. Drug response profiling of a NID1 knockdown model demonstrated differential sensitivity to HSP90 inhibition. RNA sequencing and gene set enrichment analysis between NID1^high^ and NID1^low^ phenotypes showed involvement of NID1 in mitochondrial metabolic pathways known to be enriched in LSCs. Altogether, this study highlights NID1 as a novel oncogene associated with worse EFS and metabolic LSC phenotype in AML. NID1 could serve as a biomarker and aid in further mapping LSCs to establish therapeutic strategies tackling the high relapse rates in pediatric AML.

## 1. Introduction

Using contemporary treatment protocols, five-year overall survival (OS) rates for pediatric acute myeloid leukemia (pedAML) have reached 70–75% [1,2]. Nevertheless, for some genetic subgroups, the outcome remains dismal, and chances for survival upon relapse are poor [2,3]. In addition, treatment causes a whole array of short- and-long-term toxicities. Therefore, the identification of novel therapeutic targets is warranted.

The high relapse rates in pedAML are most likely attributable to the presence of the leukemic stem cell (LSC), known to be quiescent and exert self-renewal capacities contributing to resistance to therapy [4,5]. Therefore, there is an unmet medical need to better characterize and target the LSC population to improve the event-free survival (EFS) and OS for these patients.

Previously, we conducted micro-array profiling of sorted LSCs and leukemic blasts (L-blasts) compared to their healthy counterparts, the hematopoietic stem cell (HSC) and normal myeloblast, respectively [6]. Interestingly, this allowed the identification of several LSC-specific genes as well as genes overexpressed in both the LSC and blast fractions, which might serve as potential therapeutic targets. Several of these genes have previously also been reported in a dataset of differentially expressed genes in the comparison between normal HSCs and adult AML LSCs [7].

Nidogens are well known for their structural role in the formation of the basement membrane [8,9], acting as a linker between collagen IV and laminin networks [9]. The nidogen family in humans consists of two members: nidogen-1 (NID1) and nidogen-2 (NID2). NID1 is a sulfate glycoprotein that has a role in the extracellular matrix and basement membrane in establishing a link between laminins, collagens and proteoglycans, and cell surface receptors to control cell polarization, migration, and invasion [10]. Recently, NID1 has been shown to be involved in carcinogenesis [11] and to promote migration, invasion, and chemoresistance in different tumor types [10,11,12]. Nevertheless, its role outside the context of the extracellular matrix and in leukemogenesis has not been described.

In this study, we identified NID1 expression in pedAML LSCs, while being absent in HSCs. As LSCs play a major role in the high relapse rates of AML patients, we aimed to explore the potential role of NID1 in the pathogenesis of pedAML.

## 2. Results

### 2.1. NID1 Is Expressed in the Leukemic Stem Cells and Leukemic Blasts of pedAML Patients

**Figure 1:**
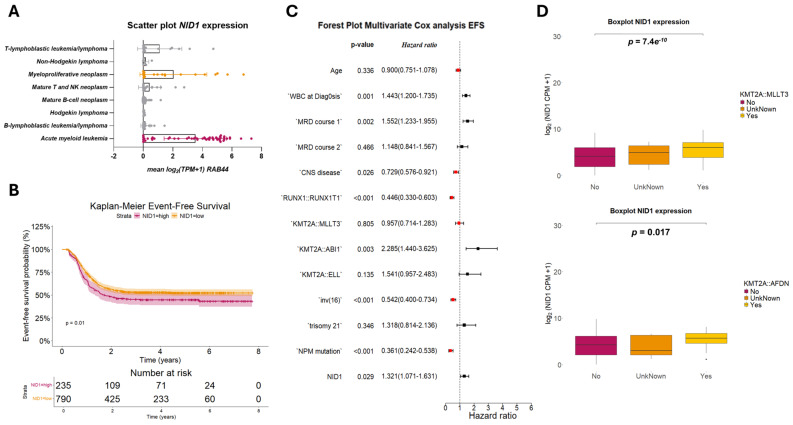
(**A**) NID1 is highly expressed in AML and myeloproliferative neoplasm cell lines and associated with poor EFS and KMT2A rearrangement in pediatric AML patients. Scatter plot of log_2_-transformed transcript per million (TPM) NID1 values plus one pseudocount for different hematological malignancies. (**B**) Kaplan–Meier curves of event-free survival according to NID1 expression in pediatric patients with AML from the TARGET AML program. (**C**) Multivariate Cox analysis showing the independent prognostic value of NID1 for EFS. (**D**) Box plots of NID1 expression, in counts per million (CPM), of two KMT2A fusions (*KMT2A::MLLT3* and *KMT2A::AFDN*) significantly associated with NID1 in patients from the TARGET AML program. FC, fold change.

By means of micro-array profiling, we identified nidogen-1 (NID1) expressed in LSCs compared to its low to absent expression in HSCs (Table S1) [6]. This was further validated in an independent set of LSC (n = 5) and HSC (n = 2) fractions by means of RT-qPCR (Figure S1A). Consulting the DEPMAP database, consisting of the gene expression data of human cancer cell lines, we observed a specific expression of NID1 in AML, myeloproliferative and few mature B-cell neoplasm cell lines compared to other hematological malignancies (Figure 1A). Interestingly, this was not observed for NID1’*s* family member nidogen-2 (Figure S1B). We validated these observations using an in-house set of hematological cell lines, by means of qPCR (n = 11) and Western blot (n = 7) (Figure S1C,D). This further confirmed the preferential expression in AML cell lines and showed that the RNA expression is in line with the endogenous protein levels of NID1. As NID1 has already been described in solid cancers as a potential regulator of metastasis and chemoresistance, we focused our further analyses on deciphering a potential functional role of NID1 in pedAML.

### 2.2. NID1 Expression Is Associated with KMT2A-Rearrangements and Worse Event-Free Survival

We investigated whether NID1 expression is associated with clinical and/or molecular parameters and whether it has value as a prognostic marker for the EFS of patients with AML. We examined NID1 expression using RNA sequencing data from pediatric patients with AML (n = associated significantly with worse EFS (*p* = 0.01) in this cohort (Figure 1B), as corroborated by multivariate Cox regression results [EFS: HR 1.321, 90% CI 1.071–1.631, *p* = 0.029; Figure 1C].

Moreover, we demonstrated that NID1 expression was associated significantly with the KMT2A-rearranged fusions *KMT2A::MLLT3* and *KMT2A::AFDN*, among four fusions included in the TARGET database (Figure 1D, Table S2). The association with KMT2A rearrangements was further confirmed using RNA sequencing data from the independent St. Jude’s cohort (n = 160), which includes 23 patients with a KMT2A rearrangement (Figure S2). Further strengthening the association, the majority of the hematological cell lines expressing NID1 harbor a KMT2A rearrangement, THP1 (*KMT2A::MLLT3*), Mono-Mac-6 (*KMT2A::MLLT3*), MV4-11 (*KMT2A::AFF1*), and KARPAS45 (*KMT2A::FOXO4*), respectively. Importantly, these associations were specific for NID1 and not for its related family member *NID2* (Table S3).

### 2.3. NID1 Modulation Alters Sensitivity Towards HSP90

**Figure 2:**
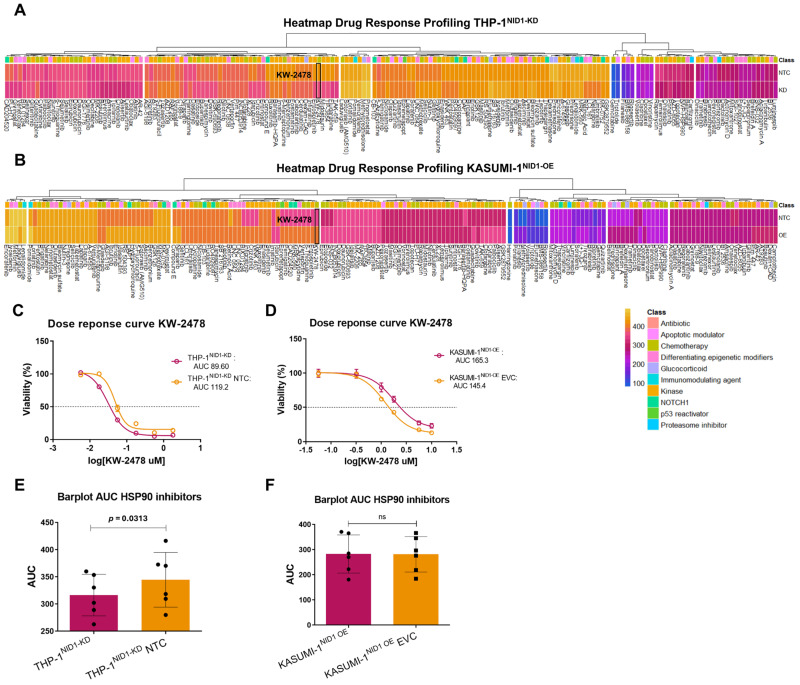
(**A**) Heatmaps of areas under the curve (AUCs) for 175 compounds measured in THP-1^NID1-KD^ and THP-1^NID1-KD^ NTC samples. (**B**) Heatmaps of areas under the curve (AUCs) for 175 compounds measured in KASUMI-1^NID1-OE^ and KASU-MI-1^NID1-OE^ EVC samples. (**C**) Dose-response curves for KW-2478 in THP-1^NID1-KD^ and THP-1^NID1-KD^ NTC samples (AUCs = 89.60 and 119.2, respectively). (**D**) Dose-response curves for KW-2478 in KASUMI-1^NID1-OE^ and KASUMI-1^NID1-OE^ EVC samples (AUCs = 165.3 and 145.5, respectively). (**E**) Dot plots of AUCs for heat shock protein (HSP) 90 inhibitor small molecules in THP-1^NID1-KD^ and THP-1^NID1-KD^ NTC samples. (**F**) Dot plots of AUCs for HSP90 inhibitor small molecules in KASUMI-1^NID1-OE^ and KASUMI-1^NID1-OE^ EVC samples.

As higher NID1 expression was shown to be associated with worse EFS, we aimed to uncover more about the biological role of NID1 in pedAML. We generated cell line models with altered NID1 gene expression by means of lentiviral shRNA knockdown in THP-1 (THP-1^NID1-KD^) and NID1 overexpression in the KASUMI-1 (KASUMI-1^NID1-OE^) cell line. Knockdown (*p* < 0.001) and overexpression (*p* = 0.001) were validated using qPCR (Figure S3A,B). To identify pathways that NID1 exploits to exert its biological function, we performed drug response profiling (DRP) consisting of 175 FDA-approved small molecules inhibitors, targeting multiple pathways, on the generated THP-1^NID1-KD^ and KASUMI-1^NID1-OE^ cell lines compared to the NTC and EVC, respectively. We used the modulated cell line models as we were mainly interested in the small molecules that showed differential sensitivity upon NID1 modulation. The DRP revealed an increased sensitivity to heat shock protein (HSP)90 inhibition in THP-1^NID1-KD^ compared to the NTC (delta AUC (AUC NTC—AUC KD) < −3 for KW-2478) and the opposite in KASUMI-1^NID1-OE^, and a decreased sensitivity for HSP90 inhibition (delta AUC (AUC EVC—AUC OE) > 3 for KW-2478) compared to the EVC (Figure 2A,B). The differential sensitivities were validated in both the KD and OE models (Figure 2C,D). Assuming HSP90 inhibitors act generally through the same mode of action, it was indeed found in THP-1^NID1-KD^ that there is a significant increased sensitivity towards HSP90 inhibitors upon NID1 knockdown compared to the NTC (Figure 2E). However, this could not be validated in KASUMI-1^NID1-OE^ (Figure 2F). The different impact of HSP90 inhibition upon NID1 modulation needs further detailed investigation.

### 2.4. NID1 Modulation Impacts Genes Enriched in Metabolic Pathways in the Leukemic Stem Cell

**Figure 3:**
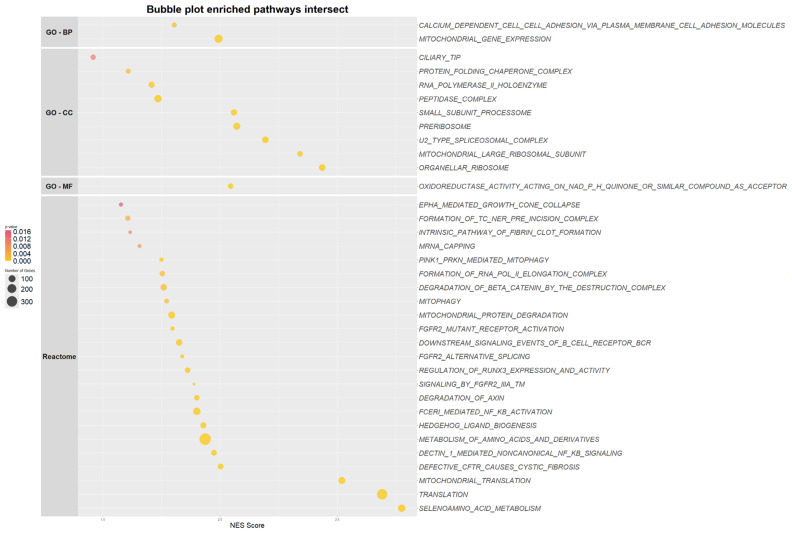
Bubble plot of enriched pathways from the reactome and gene ontology (GO)—biological processes (BP), cellular component (CC), and molecular function (MF)—databases for *KMT2A::MLLT3* NID1high pedAML patients of the TARGET database, present in the intersect with enriched pathways of the KD and OE constructs.

To identify pathways where NID1 could play a role, we performed a gene set enrichment analysis comparing the phenotypes knockdown versus NTC and overexpression versus EVC. Due to the small sample size of the expression data of the modified cell lines, it is challenging to identify significant pathways. Therefore, to identify biologically relevant pathways associated with NID1, we generated the intersection of enriched pathways in the NID1 knockdown samples, NID1 overexpression samples, and pathways that are significantly enriched (*p* value < 0.05 and FDR < 0.25) in NID1 high *KMT2A::MLLT3*-positive patients. For the latter, we selected pedAML patients from the TARGET AML program who carry a *KMT2A::MLLT3* fusion (n = 87) and ranked the patients based on their NID1 expression. From this, we selected 11 patients with high NID1 expression (above the third quartile) and 11 patients with low NID1 expression (below the first quartile) and balanced them for relapse state, OS, and central nervous system involvement (Table S4). Three-way intersecting pathways (Figure S4) involved mainly mitochondrial-linked processes, such as oxidoreductase activity, PINK1-PRKN mediated mitophagy, metabolism of amino acids, and Wnt signaling (degradation of axin and degradation of beta catenin by the destruction complex) (Figure 3).

## 3. Discussion

Despite recent advances in the OS of children with AML, still one third of the patients relapse after primary treatment [13,14]. Leukemic stem cells are thought to be implicated in the etiology. Chemoresistance, and thus in relapse of both adult and pedAML. Moreover, it is known that patients with AML expressing LSC signatures have a significantly worse prognosis, most likely attributable to the persistence of therapy-resistant cells and the self-renewal capacities of the LSC [13,15,16]. Development of LSC-targeted therapy is an important path forward to improving the EFS and OS for these patients. However, this is a challenge due to the high resemblance of targetable antigens of the LSC with the HSC [13,17]. Therefore, gaining a better understanding of LSC biology is urgently needed.

In this study, we identified that NID1 is overexpressed in the LSCs and L-blasts of pedAML patients compared to healthy cord blood HSCs and myeloblasts, suggesting that NID1 expression may be a player in the underlying pathogenesis, while the absence of expression in the HSC offers opportunities for potential NID1 targeting. Interestingly, NID1, among other genes, was significantly upregulated in the induced LSC+ fractions of the AML-induced pluripotent stem cell line model established by Wesely et al. [18]. Furthermore, our results demonstrate that NID1 expression is an independent prognostic factor for EFS in a pedAML patient cohort of the TARGET AML program. It will be of interest in future studies to evaluate in detail in larger cohorts of patients whether NID1 expression is closely linked to the LSC phenotype.

Our analyses showed that NID1 is significantly associated with the KMT2A-rearranged fusions *KMT2A::MLLT3* and *KMT2A::AFDN* in pedAML patients. KMT2A-rearranged AML is a heterogeneous pedAML subtype, and the outcome in this subtype is highly variable and related to the KMT2A fusion partner [19,20]. Adverse risk groups include patients with *KMT2A::AFF1*, *KMT2A::AFDN*, *KMT2A::MLLT3*, *KMT2A::ABI1*, and *KMT2A::MLLT1*, together representing 30% of pedAML patients [21]. KMT2A rearrangements activate a transcriptional program characterized by upregulation of *HOXA* genes and *MEIS1*, all associated with the expression of stemness markers [13]. We only found a significant association with *KMT2A::MLLT3* and *KMT2A::AFDN* fusions in the patient cohort of TARGET. However, the TARGET expression database had expression data of only four KMT2A fusion types and St Jude’s cohort only a total of 23 KMT2A-rearranged patients. We thus could not evaluate the association of NID1 expression with other KMT2A fusions. Therefore, it would be interesting to further investigate the association in a larger KMT2A-rearranged patient cohort to discern if the association is general or specific to certain fusion partner and linked biological processes. Interestingly to note, the fusions *KMT2A::MLLT3* and *KMT2A::AFDN*, which were significantly associated with NID1 and a known adverse-risk classifier, did not have a significant effect on the EFS of the TARGET pedAML cohort. This observation sheds light on a possible role of NID1 as a biomarker that may contribute to better classification of patients into high risk in the future.

By means of drug response profiling, we detected a modest significant differential sensitivity to the inhibition of HSP90, an important HSP in the context of hematological malignancies. HSP90 has been shown to control several oncogenes, including hypoxia-inducible factor (*HIF*)-*1α*, which plays a crucial role in regulating the adaptive response of leukemic cells to changes in the hypoxic environment [22,23]. Recent research indeed suggests that the rate of oxygen consumption and cell metabolism is responsible for the hypoxic nature of the BM niche where the LSCs reside [24], pushing them into a dormant state. Interestingly, in salivary gland adenoid cystic carcinoma, under hypoxic conditions, HIF-1α binds to the promotor of NID1 to upregulate NID1 expression and increases cancer cell invasion and migration via the PI3K/AKT-EMT pathway [25]. Further research is needed to evaluate whether HIF-1α is involved in the upregulated expression of NID1 in the leukemic stem cells in AML.

In addition to modulating HIF-1α levels, HSP90 has the ability to coordinate and sustain several metabolic pathways [22]. Among HSP90 homologs, the mitochondrial chaperone tumor necrosis factor receptor-associated protein 1 (TRAP1), is a key regulatory factor involved in a metabolic switch between mitochondrial respiration and aerobic glycolysis [26]. Corroborating this finding, gene set enrichment analysis of genes impacted by NID1 modulation revealed that NID1 may be involved in mitochondrial metabolic processes. Recent research has shown that LSCs and therapy-resistant cells are dependent on mitochondrial metabolism to maintain a low metabolic rate [27]. As a result, LSC are uniquely reliant on oxidative phosphorylation, executed in the mitochondria and fueled by amino acid or fatty acid metabolism, for their energy production. Active mitochondria are a major source of cellular reactive oxygen species (ROS), which are imperative for stem cell homeostasis. Therefore, to remain quiescent, LSCs actively engage mitophagy to maintain low ROS levels. The pathways reported to have a part in the metabolic phenotype in LSCs are similar to those enriched in the NID1 phenotype. Importantly, these pathways should be validated, most relevant in an in vivo setting, to fully capture the metabolic state and involvement of NID1 in AML therein. Although providing promising preliminary insights, further in-depth research is needed to fully elucidate the role of NID1 in metabolic processes in AML.

Several limitations of this study need to be acknowledged. First, validation of these associations of NID1 in KMT2A-rearranged patients in a large cohort of KMT2A-rearranged patients is needed. Importantly, only the association of four KMT2A fusions could be investigated in our study, making it unclear whether the association of NID1 with *KMT2A* is specifically linked to a particular KMT2A fusion. Furthermore, we were not able to identify an impact on HSP90 sensitivity in the overexpression model. As this could be due to the cell line genetic markup, it will be of interest to overexpress NID1 in cell lines with different molecular genetics. Lastly, gene set enrichment analysis revealed the involvement of NID1 in metabolic processes in AML. However, we acknowledge that further validation of these pathways is essential to elucidate the further role of NID1. Currently, we are investigating which strategy and models will be best suited for this.

In conclusion, this study highlights NID1 as a novel potential oncogene associated with worse OS and EFS and a metabolic LSC phenotype in AML. As the identification of unique surface antigens on the LSCs has proven to be difficult due to the high resemblance with the HSC, further research should also focus on the molecular and functional characterization of the LSC’s capacity to circumvent treatment and re-initiate leukemia. Therefore, the association with KMT2A rearrangements and the absent expression in HSC emphasizes that NID1 could serve as an interesting biomarker and help us to further map this aggressive cell population and establish potential therapeutic strategies tackling the high relapse rates in AML patients.

## 4. Materials and Methods

### 4.1. Patients and Controls

Bone marrow (BM) and/or peripheral blood (PB) from four pedAML patients with diverse molecular characteristics was selected based on cell viability (>50 × 10^6^ after routine workup) and CD34+ positivity (≥1%). Patients were diagnosed in Belgium and classified as standard risk in the protocol DB-AML-01 [28]. The characteristics of the four de novo pedAML patients used for sorting and micro-array profiling of CD34+/CD38+ and CD34+/CD38− cell fractions can be found in Table S5. As a control, three cord blood (CB) samples obtained after full-time delivery were used. All subjects gave their informed consent for inclusion before participation.

### 4.2. Micro-Array Analysis

The method used to perform micro-array profiling has been described previously in more detail [6]. In brief, the RNA of three LSC and four leukemic blast (L-blast) fractions, next to two HSC and three control blast (C-blast) controls, were profiled on a custom designed Agilent 8 × 60 K humane gene expression micro-array platform by Biogazelle. The micro-array contained probes for all human protein-coding genes (n = 27,071) and lncRNA probes (n = 30,168). Differentially expressed genes were identified based on |log_2_FC| > 2 and adjusted *p*-values (adj. *p*) < 0.05 using the limma package (R Bioconductor) [29].

### 4.3. Cell Culture

Cell lines were obtained from the German Collection of Microorganisms and Cell Cultures GmbH (DMSZ, Griesheim, Germany) and culture according to the manufacturer’s guidelines (Table S6). Culture media was supplemented with 10% or 20% heat-inactivated fetal bovine serum (Gibco™), 1 mM sodium pyruvate (Gibco™), 2 mM L-glutamine (Gibco™), penicillin (100 U/mL)-streptomycin (100 µg/mL), and amphotericin B (0.25 µg/mL) (Gibco™). Cells were maintained under 5% CO_2_ at 37 °C. Cell cultures were verified to be free of mycoplasma using the MycoAlert^®^ Mycoplasma Detection Kit (Westburg Life Sciences, Leusden, The Netherlands).

### 4.4. Public Expression Data

NID1 expression profiles of different hematological cancer cell lines were visualized using the cancer Dependency Map portal (https://depmap.org/portal/) [30]. RNA sequencing data and corresponding clinical information of pedAML patients of the Therapeutically Applicable Research To Generate Effective Treatments (TARGET) repository initiative phs000465 (https://www.cancer.gov/ccg/research/genome-sequencing/target, accessed on 23 April 2024) and the St Jude’s cohort were retrieved from the Genomic Data Commons portal (https://portal.gdc.cancer.gov, accessed on 23 April 2024) and from St. Jude’s Cloud (McLeod, 2021 (https://pecan.stjude.cloud/, accessed on 12 April 2023), respectively.

### 4.5. RNA Isolation, cDNA Synthesis and qPCR

Total RNA was extracted using the QIAzol lysis reagent (Qiagen Benelux B.V.—Antwerp, Belgium) and purified using the miRNeasy micro or mini kit (Qiagen, Inc.) according to the manufacturer’s guidelines in combination with on-column DNase I digestion using a RNase-free DNase kit (Qiagen, Inc.). RNA concentrations were measured using Nanodrop. The integrity and quality of the RNA was examined and A260/A280 ratios of ~2 were achieved. Afterwards, RNA was stored at −80 °C until further use. cDNA (200 or 400 ng) was synthesized using the PrimeScript RT Master Mix (Takara Bio Europe, Saint-Germain-en-Laye, France) according to the manufacturer’s guidelines. The reaction was established in a Veriti™ Thermal Cycler (Thermo Fisher Scientific, Merelbeke, Belgium) for 15 min at 37 °C and 5 sec at 85 °C. After cDNA synthesis, the samples were diluted to a concentration of 5 ng/µL and stored at −20 °C. A forward and reverse primer for NID1 was designed using the primer blast tool (NCBI, accession number NM_002508.3), and their efficiency was evaluated using a dilution series of pooled cDNA of human reference RNA (750500, Agilent Technologies N.V., Zaventem, Belgium) and in-house hematological cell lines (n = 20). The slope of the standard curve was −3.3178 and corresponded with an efficiency of 2.002 or 100%. qPCR reactions were carried out in a 96-well plate using Takyon™ No ROX SYBR 2× MasterMix blue dTTP (Eurogentec, Seraing, Belgium). In brief, 1 µL cDNA (5 ng/ul) was added to 9 µL of PCR mix (3.4 µL nuclease-free water, 0.3 µL forward and reverse primer (10 µM), and 5 µL Takyon™ MasterMix. Reactions were performed in a ViiA 7 Real-Time PCR system (Applied Biosystems, Thermo Fisher Scientific, Merelbeke, Belgium) using a 2-step real-time protocol (3 min 95 °C, followed by 45 cycles (15 sec 95 °C and 1 min 60 °C), combined with melting curve analysis (65 °C to 95 °C, gradually increasing at 0.1 °C/s). A minimum of two technical replicates were taken along, and a blank nuclease-free water control was taken along to examine any presence of contamination. Quantstudio™ Real-Time PCR software v1.6.1 (Thermo Fisher Scientific, Merelbeke, Belgium) was used to investigate the amplification curves and melt curve plots. The expression of NID1 was normalized against reference genes *TBP* (NM_003194) and *HPRT1* (NM_000194). Normalized relative expression values were calculated using the ΔCq-method [31]. The primer sequences for *TBP* and *HPRT1* were taken from the manuscript of Vandesompele et al. [31]. All primers were purchased at IDT technologies, and the primer sequences are listed in Table S7.

### 4.6. Western Blot Analysis

Proteins were extracted using lysis buffer containing Triton X-100 (1:100), Halt™ Phosphatase Inhibitor Cocktail (1:100, Thermo Fisher Scientific, Merelbeke, Belgium), Halt™ Protease Inhibitor (1:100, Thermo Fisher Scientific, Merelbeke, Belgium), and Dulbecco’s phosphate-buffered saline (D-PBS, Thermo Fisher Scientific, Merelbeke, Belgium). The Pierce™ BCA Protein Assay Kit (Thermo Fisher Scientific, Merelbeke, Belgium) was used to measure the protein concentration of the extracts. Next, 25 µg cell lysates were fractioned by SDS-PAGE using 10% mini-PROTEAN^®^ TGX™ Precast Protein gel (Bio-Rad Laboratories, Lokeren, Belgium.) and eventually transferred to a Trans-Blot Turbo Mini 0.2 um polyvinylidene fluoride membrane (Bio-Rad, Inc.). Primary Entactin Monoclonal Antibody (1:50,000, Proteintech, Manchester, UK.) and secondary anti-mouse IgG, HRP-linked antibody (1:10,000, Cell Signaling, Leiden, The Netherlands) were used in order to detect NID1. Protein lysates were prelabelled with Cy5 in order to perform total protein normalization. Normalized relative protein expression was calculated using ImageQuant™ TL version 11.0 analysis software (Cytiva, Marlborough, MA, USA).

### 4.7. Lentivirus Generation and Transduction

NID1 knockdown (KD) in the cell line THP-1 was established by means of lentiviral shRNAs targeting NID1 (shERWOOD UltramiR shRNA Lentiviral Target Gen Set, Transomic Technologies, Huntsville, AL, USA). NID1 overexpression (OE) in the cell line KASUMI-1 was generated using a lentiviral vector containing the open reading frame (ORF) of human NID1 (Transomic Technologies, Huntsville, AL, USA). Vector maps of both constructs can be found in Figure S1. A non-targeting vector (NTC) and an empty vector (EVC) control were included to generate the control cell lines for the KD and OE, respectively. In brief, 1.3 × 10^6^ HEK293T cells were cultured in a 6 cm dish 24 h before lentiviral packaging. The 3 µg target plasmid together with 1.5 µg envelope plasmid (pCMV-VSVG; ref.8454 Addgene, Watertown, NY, USA) and 3 µg packaging plasmid (pCMV-dR8.2; ref. 8455 Addgene, Inc.) were resuspended in 226 µL NaCl and nuclease-free water (Sigma-Aldrich, Overijse, Belgium) to a total volume of 250 µL. Subsequently, a mix of 10 µL jetPEI^®^ and 240 µL NaCl was added to the DNA plasmids and incubated for 30 min at room temperature in order to impart a net cationic charge allowing efficient transfection. Eventually, 500 µL of the DNA complex mix was transfected to the HEK293T cells and incubated for 48 h at 37 °C and 5% CO_2_. After the incubation period, the lentiviral particles were harvested and stored at −80 °C.

To transduce the cell lines of interest, 1.6 × 10^6^ cells/mL were seeded in a sterile tissue culture 6-well plate in its corresponding medium without FBS. Polybrene (250×) was added to ensure a good interaction between the cells and virus. The viral particles were added in a 1:1 ratio and the viral particles brought into close proximity with the cells by spinning the plates for 90 min at 32 °C and 2300 rpm. The cells were incubated with the viral particles for 24 h at 37 °C and 5% CO_2_. Next, the medium of the lentiviral-transduced cells was refreshed, and after another 48 h of incubation, the transduction efficiency was measured by means of a fluorescent marker present in the target vector. The transduced cells underwent selection with puromycin (2 µg/mL) until transduction efficiency reached more than 95%.

### 4.8. RNA Sequencing and Differential Expression Analysis

Total RNA of three independent replicates per condition (knockdown, non-targeting control, overexpression and empty vector control) was processed at Eurofins Genomics Europe Sequencing GmbH, Ebersberg, Germany. The samples were subjected to paired-end sequencing on the Illumina NovaSeq 6000 platform. Sequencing reads were aligned to the human genome (GRCh38.p14) using STAR alignment (STAR/2.7.6a-GCC-10.2.0). After, BAM files were subjected to featureCounts program of the Subread package (Subread/2.0.3-GCC-9.3.0). Gene counts were filtered out if the row sum of the counts in all the samples was less than 5. Subsequently, the counts were normalized, and differential expression analysis was performed using DESeq2 [32]. Raw data can be found under GSE292050 (release 13 June 2025).

Gene set enrichment analysis (GSEA) analysis was conducted with the GSEA tool of Broad Institute using the default parameters (GSEA (gsea-msigdb.org) and the reactome and gene ontology (GO) molecular function (MF), cellular component (CC) and biological process (BP)) databases.

### 4.9. Drug Response Profiling

A small molecule chemical library of 175 FDA-approved chemotherapeutic and targeted oncology compounds was used for the drug response profiling (DRP) (Table S8). The library included different classes of small molecule inhibitors, such as kinase inhibitors, differentiating and epigenetic modifiers, chemotherapeutics, apoptotic machinery, transcription factor, proteasome inhibitors, and immunomodulatory compounds active in oncological and hematological malignancies. The compounds were purchased from MedChem Express (MedChem Tronica, Sollentuna, Sweden) and Selleck Chemicals (SelleckChem, Houston, TX, USA). The compounds were dissolved in dimethyl sulfoxide (DMSO) according to the manufacturer’s instructions; DMSO was used as a negative control. Assay-ready 384-well plates were prepared dispensing each compound in four dilutions ranging from 0.1 to 10,000 nM using the ASSIST PLUS pipetting robot (INTEGRA Biosciences Co. Ltd., Shanghai, China) and stored at −80 °C. The plates were then incubated in a humidified environment at 37 °C and 5% CO_2_. Cellular viability was assessed after 72 h of drug treatment using the CellTiter-Glo ATP-based assay with the Victor X4 Multilabel Plate Reader (Perkin Elmer, Waltham, MA, USA). Output data from the cell viability assay were used as input for a self-made customized R script (RStudio R version 4.3.1, Boston, MA, USA). The script was used to normalize the result to negative control wells, generate the dose-response curves, and calculate the half-maximal inhibitory concentration (IC50) and the area under the curve (AUC) [33,34].

### 4.10. Chemosensitivity Assay

Following overnight incubation in a sterile tissue culture 96-well plate, THP-1^NID1-KD^ and NTC were treated with different concentrations (0 µM, 0.0056 µM, 0.0178 µM, 0.056 µM, 0.178 µM, 0.56 µM, and 1.78 µM) of the HSP90-inhibitor KW-2478 (Selleckchem, Inc.) for 48 h with a maximum DMSO concentration of 0.0178%. Similarly, KASUMI-1^NID1-OE^ and EVC were treated with a different concentration range (0 µM, 0.056 µM, 0.32 µM, 1 µM, 1.78 µM, 5.62 µM, and 10 µM) of KW-2478 for 48 h with a maximum DMSO concentration of 0.1% DMSO. Equal volumes of the dilutions of the compound were added to each well, and no differences were observed between the cells with or without the addition of the maximum DMSO percentage. In addition, medium and solvent control were included to control for background signaling. After the incubation, 10 µL of the cell counting kit 8 (WTS-8) reagent was added to each well and incubated for 4 h at 37 °C covered from light. Next, the absorbance was measured at 460 nm on the SpectraMax M3 (Molecular Devices, Brussel, Belgium.) with a PathCheck sensor to obtain absorbance values directly proportional to the well volumes. Dose-response curves were established by fitting the normalized absorbance values with nonlinear regression using GraphPad Prism (version 8.0.2).

### 4.11. Data Processing and Statistics

Association of NID1 expression with clinical parameters was evaluated using the online available TARGET AML data. To address the effect of sequencing depth in the different pedAML patients, counts were TMM normalized, and counts per million (CPM) values were computed from the raw RNA sequencing counts using the cpm function of the EdgeR package [35]. Boxplots were generated by means of the ggplot2 package, and a Wilcoxon test of the stat_compare_means function was performed to identify statistically significant differences between two non-parametric groups (absence or presence rearrangement).

Survival curves were estimated using the Kaplan–Meier method and compared NID1high and NID1low patient groups stratified based on a cut point by means of the maximally selected rank statistics using the surv_cutpoint function of the survminer package. The cut point provides a value that corresponds to the most significant relation with survival or EFS. Kaplan–Meier curves were established using the functions survfit and ggsurvplot of the survminer package.

The GraphPad prism was used to determine the normality and statistical differences between the KD and OE cell line models compared to the NTC and EVC by means of a Shapiro–Wilk and unpaired two-tailed t-test, respectively. After the DRP, a Wilcoxon test was performed to evaluate if the mean AUC values of drug compounds within a certain drug class differed significantly among the KD and OE and the NTC and EVC, respectively.

## Data Availability

Microarray data are available at https://www.ncbi.nlm.nih.gov/geo/ under accession GSE128103 accessed on 4 April 2023. Data from the cancer Dependency Map portal can be found at https://depmap.org/portal/, accessed on 10 April 2023. RNA sequencing data and corresponding clinical information of pedAML patients of the Therapeutically Applicable Research To Generate Effective Treatments (TARGET) repository initiative phs000465 (https://www.cancer.gov/ccg/research/genome-sequencing/target, accessed on 23 April 2024) and the St Jude’s cohort were retrieved from the Genomic Data Commons portal (https://portal.gdc.cancer.gov, accessed on 23 April 2023) and from St. Jude’s Cud (McLeod, 2021 (https://www.stjude.cloud/, accessed on 12 April 2023), respectively.

## Supplementary Materials

The following supporting information can be downloaded at: https://www.mdpi.com/article/10.3390/ijms26073011/s1.

## Abbreviations

The following abbreviations are used in this manuscript:

AML	Acute myeloid leukemia
LSCJ	Leukemic stem cell
HSC	Hematopoietic stem cell
EFS	Event-free survival
pedAML	Pediatric acute myeloid leukemia
OS	Overall survival
L-Blast	Leukemic blast
NID1	Nidogen-1
NID1	Nidogen-2
BM	Bone marrow
PB	Peripheral blood
C-blast	Control blast
TARGET	Therapeutically Applicable Research To Generate Effective Treatments
GSEA	Gene set enrichment analysis
DRP	Drug response profiling
DMSO	Dimethyl sulfoxide
IC50	Inhibitory concentration 50
AUC	Area under the curve
CPM	Counts per million
HIF-1α	Hypoxia-inducible factor 1 α
HSP90	Heat shock protein 90
TRAP1	Tumor necrosis factor receptor-associated protein 1
ROS	Reactive oxygen species
