# Supplementary material for: Nidogen-1, a Player in KMT2A-Rearranged Pediatric Acute Myeloid Leukemia
Source: Int J Mol Sci. 2025 Mar 26;26(7):3011. doi: 10.3390/ijms26073011 (PMC11988693; doi:10.3390/ijms26073011)
Supplement: Supplementary file 1 [file SupplementaryFigures_rebuttal.pdf › SupplementaryFigures_rebuttal.pdf]

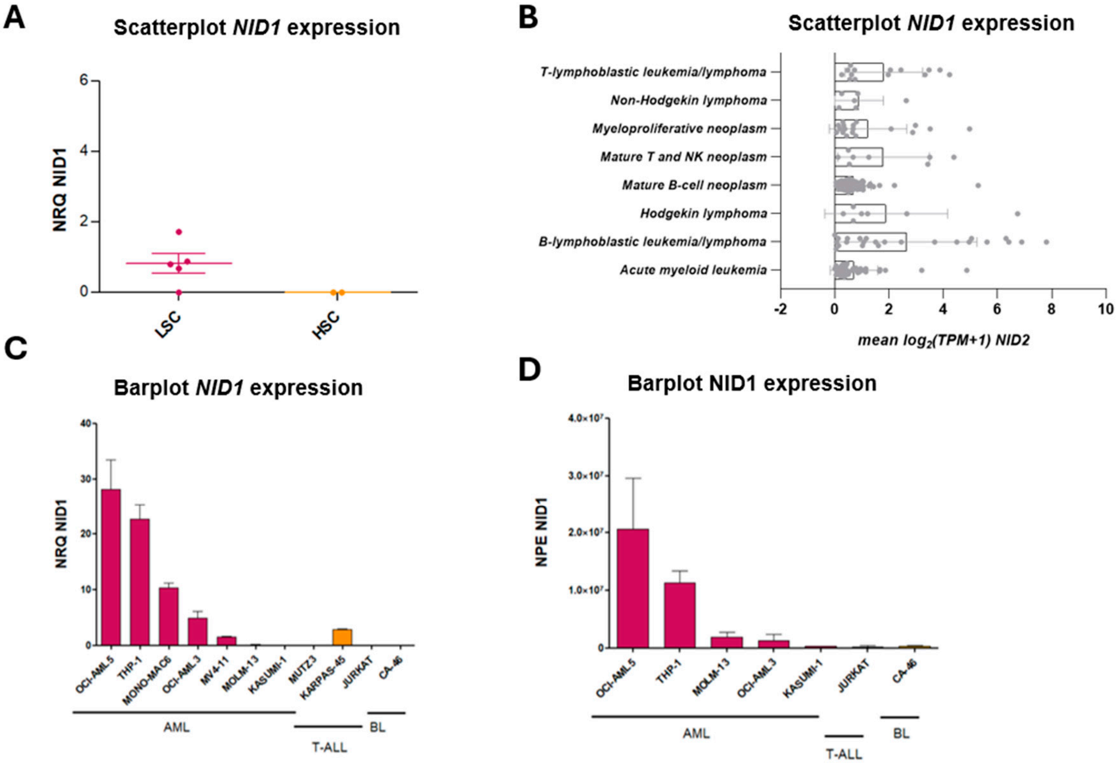

Figure S1: *Normalized relative quantities (NRQ) values of NID1 in LSC (n=5) and HSC (n=2) fractions (A); Dot plot of log2 transformed transcript per million (TPM) NID1 values plus one pseudocount for different haematological malignancies (B); NRQ values of NID1 in different acute myeloid leukemia (AML), T-cell acute lymphoblastic leukemia (T-ALL) and Burkitt lymphoma (BL) cell lines (C); Normalized relative protein values in different AML, T-ALL and BL cell lines (D).*

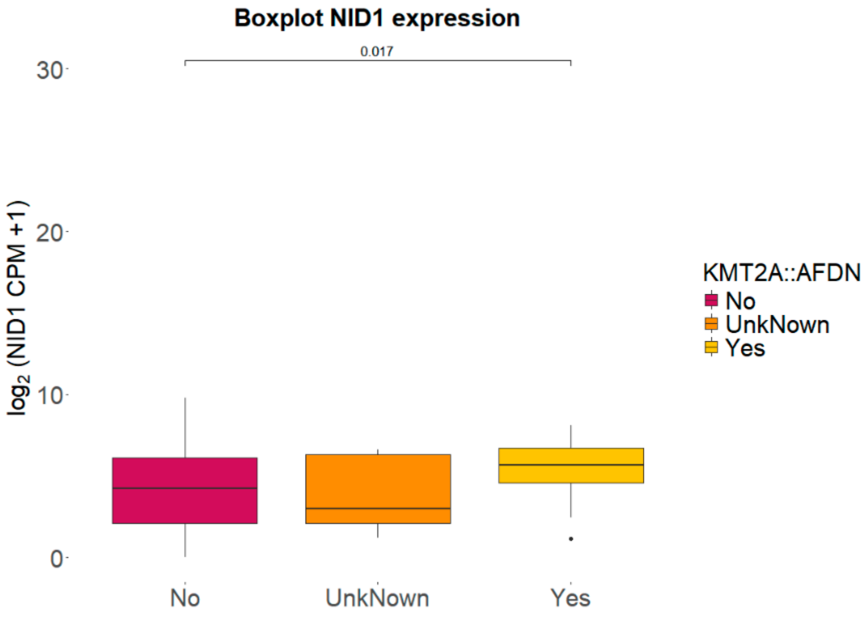

Figure S2: *Boxplot visualizing the expression of NID1 in counts per million (cpm) of pedAML patients of the St Judes cohort (n=160).*

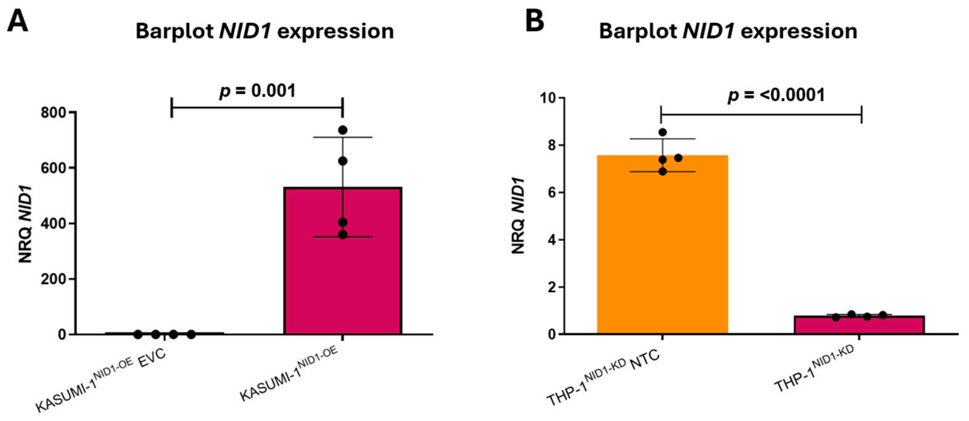

Figure S3: (a) Normalized relative quantity (NRQ) values for NID1 in THP-$1^{\text{NID1-KD}}$ and THP-$1^{\text{NID1-KD}}$ non-targeting control (NTC) samples. (**b**) Normalized relative quantity (NRQ) values for KASUMI-$1^{\text{NID1-OE}}$ and KASUMI-$1^{\text{NID1-OE}}$ empty vector control (EVC) samples.

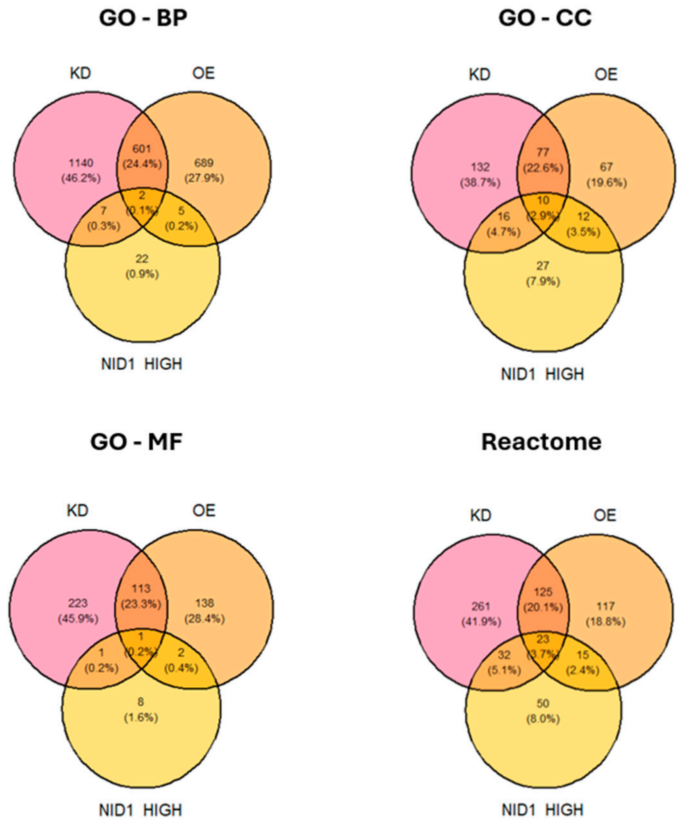

Figure S4: *Venn diagram of significant enriched pathways ((p value < 0.05 and FDR < 0.25) from the Reactome and Gene Ontology – Biological Processes (BP), Cellular Component (CC) and Molecular Function (MF) - databases for the knockdown (KD) and overexpression (OE) constructs and NID1 HIGH (KMT2A::MLLT3 $NID1^{high}$) patients of the TARGET database.*

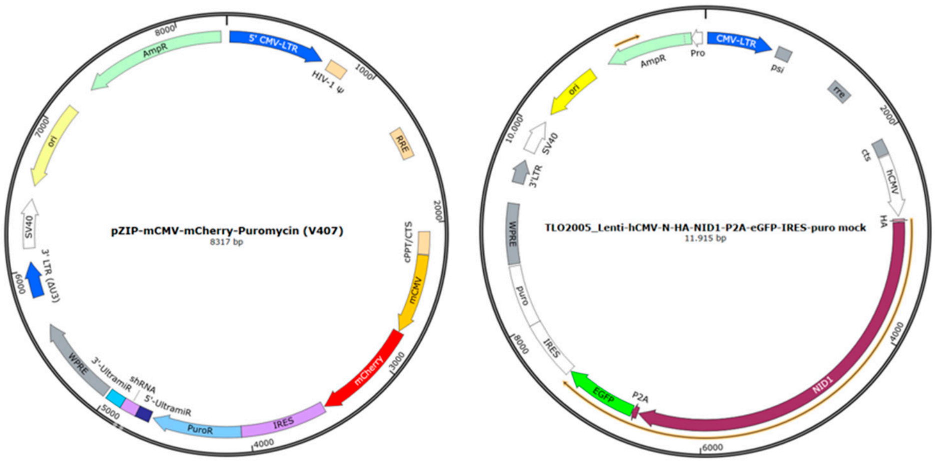

Figure S5: *Vector maps of NID1 knockdown construct (left) and NID1 overexpression construct.*
